# Coronary Computed Tomography Angiography as a Method for Diagnosing a Thrombotic Occlusion of a Giant Right Coronary Artery Aneurysm in a Patient with Difficulty in Visualizing the Right Coronary Artery During Invasive Coronary Angiography

**DOI:** 10.3390/diagnostics16101434

**Published:** 2026-05-08

**Authors:** Paweł Gać, Natalia Kusyn, Rafał Poręba

**Affiliations:** 1 Department of Environmental Health, Occupational Medicine and Epidemiology, Wroclaw Medical University, 50-367 Wroclaw, Poland; 2 Centre for Diagnostic Imaging, 4th Military Hospital, 50-981 Wroclaw, Poland; 3 Department of Biological Principles of Physical Activity, Wroclaw University of Health and Sport Sciences, 51-612 Wroclaw, Poland

**Keywords:** coronary artery aneurysm, coronary computed tomography angiography, right coronary artery

## Abstract

Giant coronary artery aneurysms, defined as those with a diameter exceeding 8 mm or a four-fold increase relative to the reference vessel segment, are incredibly rare, with an estimated prevalence of approximately 0.02% in the general population. We present computed tomography angiography images of a thrombotic occlusion of a giant right coronary artery (RCA) aneurysm. An 80-year-old Caucasian man with chronic coronary artery disease, who had undergone percutaneous coronary intervention of the middle segment of the left circumflex artery (LCx) with drug-eluting stent implantation, was referred to the computed tomography department for coronary computed tomography angiography (CCTA) due to difficulty visualizing RCA during invasive coronary angiography. In CCTA, a giant aneurysm in the proximal segment of the RCA, with a massive thrombus, communicating with the typical origin of the RCA from the right aortic bulb sinus, then extending into the occluded part of the proximal segment of the RCA, was visualised. The maximum long dimension of the RCA aneurysm was 5.3 cm, and the maximum short dimension of the RCA aneurysm was 4.4 cm. The maximum thrombus thickness in the RCA aneurysm was 2.2 cm. The middle and distal segments of the RCA, presumably filled with collateral circulation, have significantly weaker contrast, and contain numerous predominantly calcified atherosclerotic plaques. In summary, the presented CCTA images confirm the clinical importance of this modality in diagnosing coronary artery aneurysms, even in situations where the results of invasive coronary angiography remain equivocal. Due to higher spatial resolution, the ability to perform image reconstruction in multiple planes, the ability to detect thrombus, and the ability to assess the vessel wall and extracoronary structures, CCTA not only enables the detection of coronary artery aneurysms but also enables risk prediction, thus enabling the planning of a more optimal treatment strategy.

**Figure 1:**
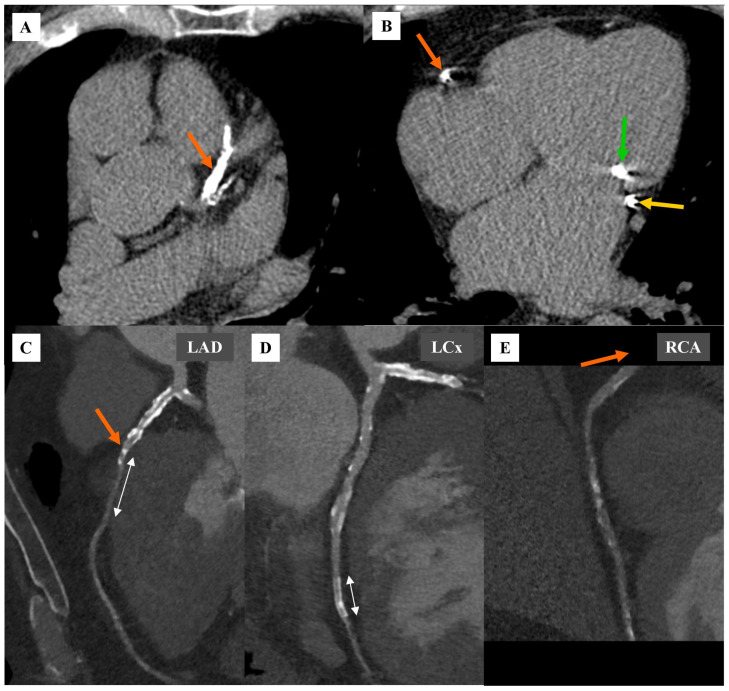
An 80-year-old Caucasian man with chronic coronary artery disease, who had undergone percutaneous coronary intervention (PCI) of the middle segment of the left circumflex artery (LCx) with drug-eluting stent (DES) implantation, was referred to the computed tomography (CT) department for coronary computed tomography angiography (CCTA) due to difficulty visualizing the right coronary artery (RCA) during invasive coronary angiography (ICA). The CCTA examination was performed using a 384-slice dual-source SOMATOM Force computed tomography scanner (Siemens Healthcare, Erlangen, Germany) using a standard protocol (described in other publication by the authors [1]). After coronary CTA, the patient is scheduled for further diagnostics and treatment at the treating center. (**A**) Native phase. Axial view. Coronary artery calcium score assessment. Massive calcifications in the left anterior descending (LAD) are marked with an orange arrow. (**B**) Native phase. Axial view. Coronary artery calcium score assessment. Massive calcifications in the LCx are marked with a yellow arrow, in the RCA with an orange arrow, and in the mitral valve with a green arrow. (**C**) Angiographic phase. Curved multiplanar reforming (cMPR) reconstruction. LAD. Multiple, predominantly calcified atherosclerotic plaques causing coronary stenosis. The orange arrow indicates stenosis of the mid-segment of the LAD artery, estimated by CT to be greater than 70%. The white marker indicates a muscular bridge in the mid-segment of the LAD artery, approximately 2.5 cm long. (**D**) Angiographic phase. Curved multiplanar reforming (cMPR) reconstruction. LCx. Multiple, predominantly calcified atherosclerotic plaques. The white marker indicates a stent in the mid-segment of the LCx artery, approximately 1.5 cm long and 0.3 cm in diameter. The stent diameter in the LCx artery was borderline for assessing its patency. No clear signs of stent restenosis were observed. (**E**) Angiographic phase. Curved multiplanar reforming (cMPR) reconstruction. RCA. The orange arrow indicates the lack of reconstruction of the proximal segment of the RCA, resulting from arterial occlusion. The middle and distal segments of the RCA, presumably filled with collateral circulation, have significantly weaker contrast, and contain numerous predominantly calcified atherosclerotic plaques, causing coronary stenosis. Assessment of the degree of stenosis is unreliable due to poor artery contrast.

**Figure 2:**
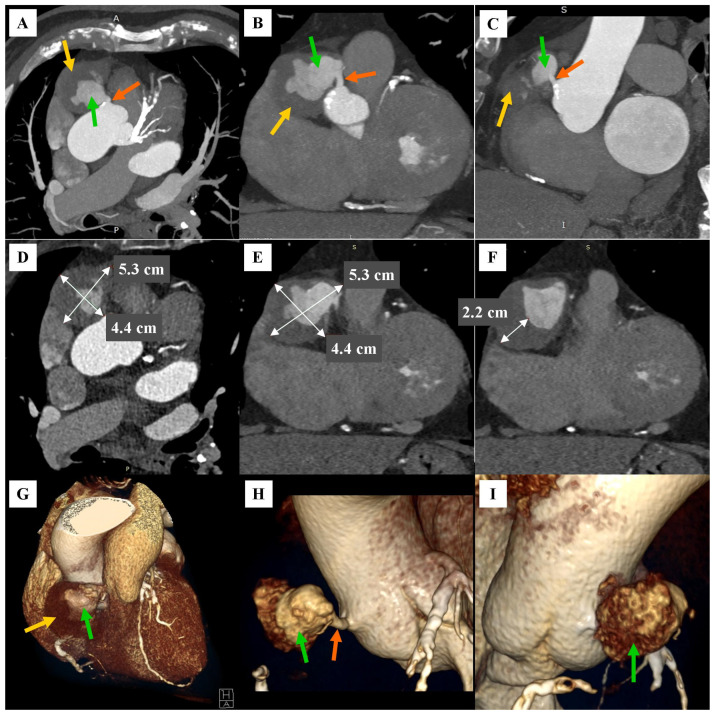
CCTA. Angiographic phase. A giant aneurysm in the proximal segment of the RCA, with a massive thrombus, communicating with the typical origin of the RCA from the right aortic bulb sinus, then extending into the occluded part of the proximal segment of the RCA. (**A**) Maximum intensity projection (MIP) reconstruction. Axial view. (**B**) MIP reconstruction. Coronal view. (**C**) MIP reconstruction. Sagittal view. In panels (**A**–**C**), the orange arrow indicates the patent part of the proximal segment of the RCA between the origin of the artery from the right aortic bulb sinus and the RCA aneurysm. The green arrow indicates the irregularly patent lumen of the RCA aneurysm. The yellow arrow indicates the thrombus within the RCA aneurysm. (**D**) Multiplanar reconstruction (MPR). Axial view at the level of the maximum dimensions of the RCA aneurysm. (**E**) MPR. Coronal view at the level of the maximum dimensions of the RCA aneurysm. In panels (**D**,**E**), the maximum long and maximum short dimensions of the RCA aneurysm are indicated by white markers. In both views, the maximum long dimension of the RCA aneurysm was 5.3 cm, and the maximum short dimension of the RCA aneurysm was 4.4 cm. (**F**) MPR. Coronal view at the level of maximum thrombus thickness in the RCA aneurysm. The white marker indicates the maximum thrombus thickness in the RCA aneurysm, which was 2.2 cm. (**G**) Volume rendering technique (VRT) reconstruction. Coronal view. The green arrow indicates the irregularly patent lumen of the RCA aneurysm. The yellow arrow indicates the thrombus within the RCA aneurysm. (**H**) VRT reconstruction with an increased cut-off point for the density of voxels included in the reconstruction. Oblique view of the RCA origin from the right aortic root sinus from the front. The orange arrow indicates the patent part of the proximal segment of the RCA between the origin of the artery from the right aortic bulb sinus and the RCA aneurysm. The green arrow indicates the irregularly patent lumen of the RCA aneurysm. (**I**) VRT reconstruction with an increased cut-off point for the density of voxels included in the reconstruction. Oblique view of the RCA origin from the right aortic root sinus from the posterior. The green arrow indicates the irregularly patent lumen of the RCA aneurysm.

**Figure 3:**
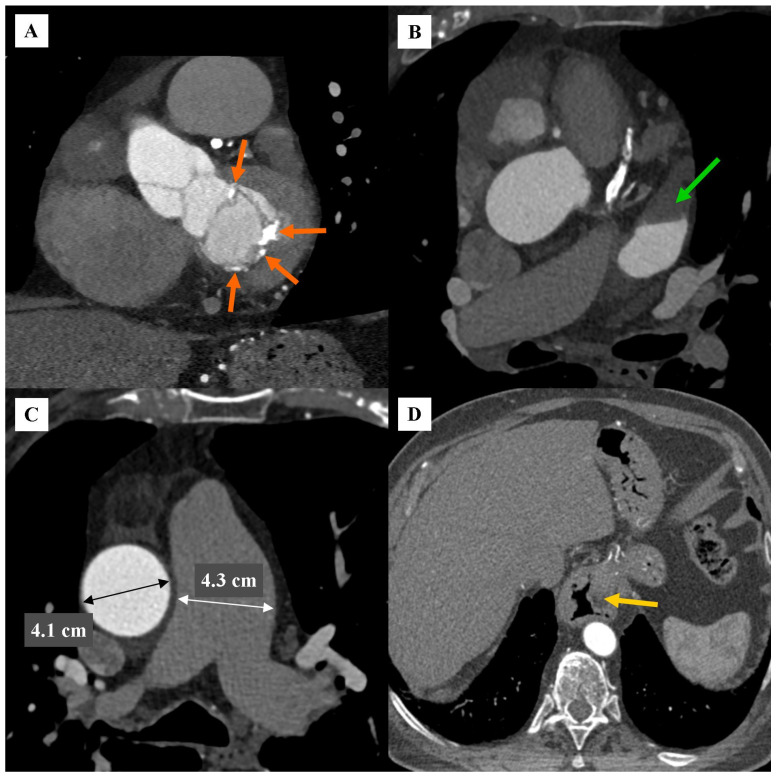
CCTA. Angiographic phase. Extracoronary and extracardiac tomographic changes. (**A**) MPR. Oblique view, in the plane of the mitral valve. Orange arrows indicate multiple calcifications in the mitral valve annulus. (**B**) MPR. Axial view. The green arrow indicates a lack of contrast in the left atrial appendage, which may correspond to a thrombus in the left atrial appendage. (**C**) MPR. Axial view. The white marker indicates the diameter of the dilated main pulmonary artery (MPA), which was 4.3 cm. The black marker indicates the diameter of the dilated tubular part of the ascending aorta, which was 4.1 cm. (**D**) MPR. Mediastinal window. Axial view. Yellow arrow indicates a sliding hiatal hernia. **Commentary:** Coronary artery aneurysm (CAA) is defined as a focal dilation of the coronary artery, which should be at least 1.5 times the width of the neighbouring normal vasculature [2,3,4]. Coronary artery aneurysms (CAAs) are a relatively uncommon clinical entity, typically identified incidentally during diagnostic imaging. The reported incidence of CAA varies between 0.3% and 5.3% among patients undergoing coronary angiography [3,5]. There is a significant gender predilection, with the condition being approximately 2 to 3 times more prevalent in men than in women [2,4,5]. RCA is the most frequently involved vessel, accounting for 40% to 70% of all reported cases, followed by LAD and LCx arteries [4,5,6]. Giant coronary artery aneurysms, defined as a diameter exceeding 8 mm or a four-fold increase relative to the reference vessel segment, are incredibly rare, with an estimated prevalence of approximately 0.02% in the general population [4,5]. In terms of gender and location, the presented case of giant coronary artery aneurysm is typical. The coexistence of a very large aneurysm with a massive intraluminal thrombus and occlusion of the RCA segment behind the aneurysm, as well as the simultaneous topography of the aneurysm resulting in an equivocal image on invasive coronary angiography, which prompted the addition of CCTA, are innovative aspects of this case. There are few published case reports in the literature describing the coexistence of giant aneurysms with massive intraluminal thrombus and occlusion of the posterior RCA segment, only a few of which are available in open access. Fanai et al. describe a case of a giant LAD aneurysm diagnosed by invasive coronary angiography and a giant RCA aneurysm with occlusive thrombus diagnosed by subsequent CCTA [7]. The RCA aneurysm measured approximately 8.0 cm in diameter, was oval, and was filled with a circular thrombus of increasing thickness. The patient did not consent to invasive treatment. As in our case, the lack of follow-up is a limitation of this publication. Gulcu et al. published a case report in Turkish [8]. In this case, the RCA aneurysm was approximately 1.5 cm in size, and a thrombus within the aneurysm occluded the vessel, causing symptoms of acute coronary syndrome. The patient was treated with tissue plasminogen activator. Ten et al. [9] and Chu et al. [10] described large multiple coronary artery aneurysms, but in a population of children with Kawasaki disease. Ten et al. described RCA thrombus occlusion in one of the RCA aneurysms measuring up to approximately 1.0 cm, treated with mechanical thrombectomy. Chu et al. described giant bilateral axillary artery aneurysms with left complete obstructive thrombus in patients with concomitant coronary artery aneurysms in children with Kawasaki disease. In the study by Keyser et al. [11], multiple coronary artery aneurysms were diagnosed, including giant LAD and RCA aneurysms. A thrombus in the RCA aneurysm caused vessel occlusion and symptoms of acute coronary syndrome. The aneurysm measured approximately 10.5 cm on computed tomography; however, the computed tomography images were not included in the publication. The patient underwent cardiac surgery, including resection of the RCA aneurysm and CABG. The development of CAA is driven by the progressive weakening of the arterial wall [2,3,4,5]. The process involves chronic inflammation, which triggers the release of matrix metalloproteinases and other proteolytic enzymes. These factors degrade the tunica media’s elastic fibers and collagen, resulting in a weakened wall that expands under intraluminal pressure [2,3,4]. Additionally, there is a rising incidence of iatrogenic CAAs resulting from PCI [2,3,5]. The prognosis of patients with CAA is highly variable and depends on different factors, such as the aneurysm’s morphology, diameter, and the presence of underlying obstructive coronary artery disease [2,3,5]. While many CAAs remain clinically silent, they are associated with several life-threatening risks. Significantly slower blood flow in the aneurysms area may cause intraluminal thrombosis, which can lead to acute myocardial infarction, sudden cardiac death and angina pectoris even in the absence of atherosclerotic stenosis [4,5]. Moreover, CAAs are at risk for spontaneous rupture resulting in fatal cardiac tamponade, as well as mechanical compression of cardiac chambers which can lead to congestive right heart failure, or the formation of heart cavities fistulas [4]. Recent evidence suggests that patients with CAA have higher rates of major adverse cardiovascular events compared to those with normal coronary anatomy, likely due to chronic inflammatory processes and unfavourable hemodynamics [2,4,5]. In the discussed case, the large dimensions of the aneurysm, massive thrombus in the lumen of the aneurysm, and RCA occlusion behind the aneurysm clearly indicate a high-risk phenotype. Detection (on CCTA) of a large right coronary artery aneurysm with a large thrombus within the aneurysm requires a more individualized approach to further patient management than typical coronary artery disease [12]. Full anticoagulation should be considered, including the use of both antiplatelet agents and anticoagulation. A more cautious approach should be taken to potential RCA angioplasty. Once myocardial ischemia in the RCA vascularization is confirmed, delayed PCI or coronary artery bypass grafting (CABG) should be considered. In the case of PCI, the use of covered stents is preferred. The patient requires more frequent follow-up and more thorough monitoring. The patient requires more rigorous control of risk factors (blood pressure, cholesterol concentrations, blood glucose concentration) as well as more frequent imaging tests [12]. The indication for CCTA in this case was difficulty visualizing the right coronary artery in invasive coronarography. Failure to visualize RCA on ICA may have several causes [13,14]. The most common include RCA origin occlusion, ectopic RCA origin, angular RCA origin, RCA spasm, and congenital absence of the RCA. None of these causes were present in the case discussed. The RCA origin was typical. However, the proximal RCA contained a large aneurysm with a massive thrombus, causing RCA occlusion behind the aneurysm. Invasive coronary angiography is a luminographic examination. The large thrombus in the aneurysm did not contrast during ICA. In this case, the patent portion of the aneurysm filled as an irregular space with turbulent inflow and no visible outflow to the periphery. The location of the aneurysm in invasive coronary angiography projections combined the above contrasting space with the aortic root, which was likely interpreted as a lack of visualization of the RCA. CCTA offers advantages over invasive coronary angiography in these circumstances. In addition to the coronary artery lumen, it also demonstrates the vessel wall and extracoronary structures. CCTA has higher spatial resolution than ICA. CCTA allows for the acquisition of multiple cross-sections without the need for increased ionizing radiation dose or the volume of contrast agent administered [14,15,16]. In this case, CCTA enabled the visualization of both the thrombus within the aneurysm and the spatial relationship of the aneurysm to the aortic root, the remaining RCA segments, and other anatomical structures. The differential diagnosis of a coronary artery aneurysm should include a pseudoaneurysm, vessel remodeling after PCI, and other structural abnormalities, such as coronary artery ectasia, coronary artery dissection, coronary artery perforation, and hematoma [4]. When differentiating between a true aneurysm and a pseudoaneurysm on CCTA, the differentiating criteria include the integrity of the vessel wall, the external shape of the aneurysm, the presence of a neck, and the location of the contrasted blood [17]. In a true aneurysm, the vessel wall is intact, the external shape of the vessel is regular, and the contrasted blood is found within the vessel lumen. A true aneurysm lacks a neck connecting the aneurysm to the native vessel. Differentiating a true aneurysm from vessel remodeling after PCI can be difficult in small aneurysms [4]. Tomographic features suggestive of a true aneurysm include a large aneurysm diameter (especially a large ratio of the aneurysm dimensions to the native vessel diameter), clear boundaries between the normal-width vessel segments and the aneurysm, a sac-shaped aneurysm, and the presence of a thrombus within the aneurysm. In our case, the images met the criteria for a true right coronary artery aneurysm. A significant limitation of the current article is the lack of images from the initial invasive coronary angiography, which failed to visualize the right coronary artery. Another significant limitation is the lack of full documentation of the diagnostic and therapeutic procedure performed so far (including the lack of information on the verification of ischemia in the right coronary artery myocardial vascularization area using stress-echo or SPECT examinations), as well as information on further treatment (after coronary CTA, the patient is scheduled for further diagnostics and treatment at the treating center). In summary, the presented CCTA images confirm the clinical importance of this modality in diagnosing coronary artery aneurysms, even in situations where the results of invasive coronary angiography remain equivocal. Due to higher spatial resolution, the ability to perform image reconstruction in multiple planes, the ability to detect thrombus, and the ability to assess the vessel wall and extracoronary structures, CCTA not only enables the detection of coronary artery aneurysms but also enables risk prediction, thus enabling the planning of a more optimal treatment strategy.

## Data Availability

The original contributions presented in this study are included in the article. Further inquiries can be directed to the corresponding author.

## Abbreviations

The following abbreviations are used in this manuscript:

CABG	coronary artery bypass grafting
CCA	coronary artery aneurysm
CCTA	coronary computed tomography angiography
cMPR	curved multiplanar reforming
CT	computed tomography
DES	drug-eluting stent
ECG	electrocardiography
ICA	invasive coronary angiography
LAD	left anterior descending
LCx	left circumflex
MIP	maximum intensity projection
MPA	main pulmonary artery
MPR	multiplanar reconstruction
PCI	percutaneous coronary intervention
RCA	right coronary artery
VRT	volume rendering techniques
